# Gut microbiota and sepsis: mechanisms, clinical correlations, and therapeutic prospects

**DOI:** 10.3389/fmed.2026.1793041

**Published:** 2026-03-23

**Authors:** Jinmin Yu, Xiaoqing Liu

**Affiliations:** 1 Emergency Department, Chongqing University Fuling Hospital, School of Medicine, Chongqing University, Chongqing, China; 2 Pediatric Department, Chongqing University Fuling Hospital, School of Medicine, Chongqing University, Chongqing, China

**Keywords:** dysbiosis, fecal microbiota transplantation, gut microbiota, immune dysregulation, sepsis

## Abstract

Sepsis is a life-threatening organ dysfunction triggered by a dysregulated host response to infection. According to the Global Burden of Disease Study, this condition affects over 50 million people annually and causes approximately 5.3 million deaths, with fatality rates varying significantly across populations and healthcare settings, ranging from about 20% to 50%, representing a major challenge in critical care medicine. In recent years, the gut microbiota, as the largest microbial ecosystem in the human body, has increasingly demonstrated a central role. It is not only essential for maintaining intestinal barrier integrity, immune homeostasis, and metabolic balance but also actively participates in the pathogenesis, progression, and outcomes of sepsis through modulating immune responses, influencing the production of key metabolites, and mediating gut-organ axes. This article systematically reviews the characteristics of sepsis-induced gut microbiota dysbiosis, delves into the molecular mechanisms by which dysbiosis drives immune disorders, metabolic disturbances, and multi-organ injury, evaluates the clinical potential and current limitations of microbiome-associated biomarkers, and summarizes recent advances and controversies in microbiota-targeted therapeutic strategies, including probiotics, fecal microbiota transplantation, precision nutrition, and antibiotic stewardship. This review aims to analyze the shortcomings and translational challenges in current research, providing a theoretical basis and forward-looking perspective for developing precise microbiome-based individualized management strategies for sepsis.

## 1 Introduction

Sepsis management has entered the “Sepsis-3” era, in which sepsis is defined as life-threatening organ dysfunction caused by a dysregulated host response to infection (1). The evolution from Sepsis 1.0 to 3.0 over more than three decades reflects an emphasis on the critical role of the host response and has propelled research into immune dysregulation mechanisms.

The global incidence of sepsis continues to rise, particularly among the elderly, immunocompromised individuals, and patients admitted to intensive care units (ICUs). Despite advances in antimicrobial therapy, fluid resuscitation, and organ support, sepsis remains associated with high mortality, frequent readmissions, and long-term functional impairments (2). Numerous studies have confirmed the correlation between high mortality rates and excessive antibiotic use as well as prolonged treatment duration (3–5). According to the Global Burden of Disease Study, sepsis contributes directly or indirectly to approximately 5.3 million deaths annually, making it one of the leading causes of global mortality (6), Current therapeutic approaches face significant limitations, including antimicrobial resistance, the inability to reverse immunoparalysis, and the lack of effective interventions for multiple organ dysfunction syndrome (MODS). In the clinical management of sepsis, the early empirical application of broad-spectrum antibiotics is closely associated with the exacerbation of antimicrobial resistance (AMR). Studies indicate that prolonged use of broad-spectrum antibiotics exhibits a positive correlation with the risk of drug-resistant bacterial infections. Inappropriate antibiotic use not only amplifies the selective pressure for multidrug-resistant organisms (MDROs) but also disrupts the patient’s intestinal microbiota, thereby promoting the dissemination of resistance genes (7–9).

A recent systematic review concluded that existing clinical interventions have failed to significantly improve sepsis-related mortality (10). A significant source of challenges in sepsis clinical research lies in the prevalent adoption of a “one-size-fits-all” model in trial design. Sepsis exhibits marked clinical heterogeneity (11, 12). When diverse pathogens, including bacteria, viruses, and fungi, interact with host-specific factors such as genetic background, environmental exposures, and chronic comorbidities to elicit differential host responses, uniform therapeutic regimens are unlikely to achieve optimal efficacy (13). Furthermore, a study comparing risk profiles among sepsis patients of different age groups revealed that risk factors in elderly patients were primarily reflected in histories of chronic conditions such as hypertension and hyperglycemia, whereas non-elderly patients showed stronger associations with chronic renal failure and hematologic malignancies. This complex heterogeneity underscores the imperative for personalized therapeutic strategies. Therefore, novel therapeutic strategies are urgently needed (14).

The role of the gut microbiota in critical illnesses including sepsis and systemic inflammatory responses has long been established (15). The gut serves not only as the primary site for nutrient absorption and immune defense but also as the largest reservoir of microorganisms in the human body. Under conditions such as physiological stress, intestinal ischemia, or antibiotic exposure, gut barrier integrity is compromised, facilitating bacterial translocation and endotoxin leakage into the systemic circulation, thereby triggering a systemic inflammatory storm and remote organ injury. Recent advances in high-throughput sequencing and multi-omics technologies have greatly facilitated the investigation of gut microbiota in sepsis pathophysiology (16, 17). For decades, sepsis has been framed primarily as a battle against invading pathogens—an external threat to be eradicated with antibiotics and supportive care. The gut microbiota reframes this narrative, recognizing dysbiosis not as a secondary consequence but as a causal hub shifts the therapeutic focus from pathogen-centric eradication to host-microbe ecosystem restoration.

Herein, we integrate current research on the gut microbiota in sepsis, to elucidate how dysbiosis drives disease progression and to evaluate emerging microbiome-targeted therapeutic approaches, offering new perspectives for precision critical care medicine. Moreover, the convergence of metagenomics, metabolomics, and artificial intelligence now enables a level of patient stratification previously unattainable, advancing sepsis management toward precision medicine.

## 2 Structural and functional basis of the gut microbiota

### 2.1 Factors shaping bacterial communities in the host intestine

The gut microbiota is a complex microbial community that begins to establish at birth, evolves with age, and varies according to geography, diet, and host genetics (18). Numerous studies have indicated that the composition of the gut microbiota is associated with ethnicity and host genotype. Infants of East Asian descent exhibit higher abundances of *Bifidobacterium* and *Lactobacillus*, whereas the gut microbiota of Caucasian infants is predominantly characterized by bacteria of the order *Clostridiales* (19). Brooks et al. (20) analyzed data from nearly 1,700 individuals across four ethnic groups and confirmed that ethnicity is an independent factor influencing gut microbial composition. Research has also demonstrated that the gut microbiota is more similar among family members than among unrelated individuals, with monozygotic twins showing significantly higher similarity than dizygotic twins (21). Through genome-wide association studies (GWAS), Davenport et al. (22) identified at least eight bacterial taxa whose abundances are significantly correlated with human genetic variation, thereby establishing a close relationship between the composition of the gut microbiota and genetic factors.

The gut microbiota undergoes dynamic evolution throughout the human life cycle, with its diversity and structure changing regularly with age. Host age and sex collectively shape microbiota characteristics, microbial richness gradually increases from infancy to adulthood (23), while the microbiota structure readjusts in old age, with centenarians exhibiting even higher diversity (24). Meanwhile, sex differences regulated by sex hormones emerge after puberty and are associated with disease susceptibility (25), revealing the biological basis of physiological traits in microbiota evolution.

Diet plays a critical role in shaping the gut microbiota, as different dietary patterns significantly influence the composition and function of the gut microbiota, thereby affecting host health. Human milk oligosaccharides metabolized by specific bacteria produce short-chain fatty acids (SCFAs), which enhance the infant’s immune system (26). Zmora et al. (27) indicated that dietary components can be directly broken down by bacteria such as *Bacteroides* and *Bifidobacterium* into SCFAs, thereby promoting the proliferation of beneficial bacteria. Diet can also indirectly regulate the microbiota, as exemplified by microbiota imbalance resulting from vitamin A deficiency, while SCFAs help maintain immune homeostasis and intestinal barrier function by activating G-protein-coupled receptors (GPR41/43).

Antibiotics disrupt the balance of the gut microbiota while eliminating pathogenic bacteria, leading to reduced microbial diversity and upregulation of antibiotic resistance genes, with effects that may persist long-term (28). Interactions with diet and host metabolites further alter the susceptibility and recovery capacity of the microbiota to antibiotics (29), and different antibiotics exert specific effects on the microbiota. The use of fluoroquinolones reduces Gram-negative bacilli, whereas cephalosporins suppress *Enterobacteriaceae* while promoting the proliferation of *Enterococcus* (30).

Geographical location significantly influences the composition of the gut microbiota, with populations from different regions exhibiting distinct microbial diversity and structure (31). Zhang et al. (32) found that geographical location markedly affects gut microbiota composition, and the more refined the geographical division, the more significant its impact. Geographical distance is negatively correlated with microbiota similarity—the greater the distance, the more divergent the microbiota.

Gut microbiota dysbiosis is closely associated with the occurrence and development of various diseases, with mechanisms involving multiple dimensions such as immune regulation, metabolite synthesis, and maintenance of intestinal barrier function (33). Dysbiosis can trigger metabolic diseases (type 2 diabetes, obesity), autoimmune diseases (inflammatory bowel disease, rheumatoid arthritis), neurological disorders (such as autism, Parkinson’s disease), and cardiovascular diseases, among others. Nie et al. (34) identified a novel microbially derived bile acid, 3-succinylated cholic acid (3-sucCA), which enriches the beneficial bacterium *Akkermansia muciniphila*, enhances intestinal barrier function, inhibits TLR4-mediated hepatic inflammation, and significantly alleviates the progression of metabolic dysfunction-associated steatohepatitis (MASH). Takeuchi et al. (35) demonstrated that specific microbial communities can improve host insulin resistance by modulating carbohydrate metabolism. Wang et al. (36) found that microbiota-derived DPP4, under conditions of impaired gut barrier function, induces glucose intolerance. Li et al. (37) revealed that *Oscillibacter* regulates lipid homeostasis by metabolizing various types of cholesterol, thereby contributing to reduced cardiovascular disease risk.

### 2.2 Composition and function of the host gut microbiota

In healthy adults, the gut microbiota is predominantly composed of members of the phyla *Bacteroidetes* and *Firmicutes*. At the genus level, beneficial taxa commonly include *Bifidobacterium*, *Lactobacillus*, *Prevotella*, and *Faecalibacterium*. Notably, *Faecalibacterium* is a major producer of short-chain fatty acids (SCFAs), particularly butyrate, which exerts anti-inflammatory effects and plays a vital role in host health (38). Beyond bacteria, the intestinal ecosystem also encompasses fungi (the mycobiome) and viruses (the virome), which are integral to microbial community structure and host physiology. Commensal fungi, such as various Candida species, contribute to immune modulation and metabolic balance, interacting with bacteria to influence intestinal homeostasis and susceptibility to infection (39). Concurrently, the intestinal virome—particularly bacteriophages—regulates bacterial population dynamics, facilitates horizontal gene transfer, and modulates microbial metabolism, thereby influencing both microbial stability and host-microbe interactions (40).

In addition to these diverse microbial constituents, the gut microbiota as a whole interacts closely with the host immune system to maintain immune tolerance, prevent excessive inflammatory responses against self-tissues and commensals, and strengthen the host’s defense against external pathogens. The gut microbiota and its metabolites engage in extensive crosstalk with the host through multiple mechanisms. In terms of barrier maintenance, the microbiota enhances mucus secretion by goblet cells and upregulates the expression of tight junction proteins, thereby strengthening epithelial integrity and inhibiting pathogen colonization (41). Probiotics enhance the expression of tight junction proteins to reduce intestinal permeability, thereby maintaining barrier integrity (42, 43), and activate the epidermal growth factor (EGFR) signaling pathway through secretion of the p40 protein, promoting intestinal epithelial cell proliferation while inhibiting apoptosis, thus repairing the damaged intestinal barrier (44, 45). Furthermore, probiotics compete with pathogens for nutrients and colonization sites, and interact with the host and commensal microbiota to synergistically enhance intestinal defense function (46, 47).

Metabolically, the gut microbiota decomposes indigestible carbohydrates through anaerobic fermentation, producing short-chain fatty acids (SCFAs). SCFAs are crucial for maintaining intestinal health and overall metabolic balance by enhancing the intestinal mucosal barrier function, regulating immune responses, and suppressing inflammation. Butyrate enhances the intestinal barrier by promoting the expression of mucins and tight junction proteins. It can also act as a signaling molecule to activate G protein-coupled receptors (GPCRs) (48–50) and inhibit histone deacetylase (HDAC), thereby driving the differentiation of regulatory T cells (Treg), suppressing the release of pro-inflammatory factors (51), and regulating neutrophil function by inhibiting neutrophil migration and neutrophil extracellular trap (NET) formation, alleviating intestinal inflammation, and thus establishing a microenvironment conducive to immune tolerance (52).

Furthermore, microbe-associated molecular patterns (MAMPs) are a class of highly conserved structures present in microorganisms, such as lipopolysaccharide, peptidoglycan, and flagellin, which can be recognized by host cells through pattern recognition receptors (PRRs), such as Toll-like receptors (TLRs) and NOD-like receptors (NLRs). MAMPs can activate TLRs signaling pathways, thereby regulating the release of hormones such as cholecystokinin (CCK), incretin (GLP-1), and 5-hydroxytryptamine (5-HT) (53). A high-fiber diet favors the colonization of certain gut microbiota, which can activate the stimulator of interferon genes (STING), thereby promoting the production of type I interferons in the tumor microenvironment, effectively modulating the composition of mononuclear macrophages in tumors, improving the tumor microenvironment, and enhancing the efficacy of immune checkpoint blockade (ICB) (54). In a melanoma mouse model study, researchers found that SagA enzyme secreted by *Enterococcus* spp. in the gut releases peptidoglycan fragments from the bacterial cell wall, activating macrophages in the tumor microenvironment through the NOD2 receptor and enhancing the efficacy of immunotherapy (55).

## 3 Sepsis-induced gut dysbiosis

### 3.1 Clinical and experimental evidence of microbial alterations

Numerous clinical studies have demonstrated significant gut dysbiosis in septic patients (56–58), characterized by a marked reduction in α-diversity (reflecting species richness and evenness), and a profound imbalance in the Firmicutes/Bacteroidetes (F/B) ratio (59–61). Opportunistic pathogens such as *Escherichia coli*, *Klebsiella*, and *Enterococcus* spp. are markedly enriched, while beneficial SCFA-producing commensals particularly *Faecalibacterium prausnitzii* and *Bifidobacterium* (56) are significantly depleted (62). These microbial shifts correlate strongly with disease severity and mortality risk and may serve as prognostic indicators. Sun et al. (63) enrolled 38 septic patients and 19 healthy controls from an ICU in a Shanghai hospital in China, analyzing fecal samples collected at admission using microbiome and untargeted metabolomics approaches. The study found that septic patients exhibited enrichment of *Enterococcus* and a reduction in beneficial bacteria; the abundance of the *Bacteroides* genus (particularly *Bacteroides vulgatus*) was positively correlated with ICU length of stay and the risk of complications. Freedberg et al. (64) found that intestinal dominance by *Enterococcus*, particularly colonization by vancomycin-resistant *Enterococcus* (VRE), was an independent risk factor for death or all-cause infection in septic patients. Liu et al. (65) included 64 patients with sepsis or septic shock and identified two enterotypes (ICU E1 and ICU E2). Compared to ICU E2, which was dominated by the *Enterococcus* genus, they found that ICU E1, characterized by a predominance of *Bacteroides*, was more likely to develop septic shock. Tang et al. employed a bidirectional two-sample Mendelian randomization approach, based on the OpenGWAS database, to investigate the causal relationship between gut microbiota and sepsis. The results indicated that the class *Lentisphaeria* and the order *Victivallales* had a protective causal association with sepsis, while the *Eubacterium eligens* group was positively associated with sepsis risk; reverse MR suggested that sepsis might influence the abundance of genera such as *Odoribacter* and *Phascolarctobacterium* (66).

Animal models corroborate these findings. In murine cecal ligation and puncture (CLP)-induced sepsis, the gut microbiota similarly exhibits reduced diversity, loss of symbionts, and overgrowth of potential pathogens (67). Sun et al. further confirmed in a rat CLP sepsis model that *Enterococcus* and *Bacteroides* participate in the pathological process by differentially regulating intestinal inflammation-related genes (e.g., Mmp9, Cxcr4), suggesting that specific microbial communities and their metabolic pathways may serve as potential targets for sepsis prognostic warning and therapy (63). The degree of dysbiosis correlates positively with systemic inflammatory markers, organ dysfunction scores, and mortality (68). Prospective studies indicate that patients with Clostridium difficile infection (CDI) have a 65%–70% increased risk of subsequently developing sepsis, suggesting that gut microbiota disruption is not merely a bystander effect but an active contributor to sepsis pathogenesis (69, 70). Collectively, these findings underscore that the gut microbiota is not a passive reflector but an active participant in the pathophysiological cascade of sepsis. The consistent association between specific microbial signatures (e.g., loss of diversity, enrichment of pathobionts like *Enterococcus*, depletion of SCFA-producers like *Faecalibacterium*) and adverse clinical outcomes positions the gut microbiota as a promising reservoir of biomarkers for early risk stratification and prognosis prediction. Integrating microbial markers with existing clinical scores could enhance the precision of early sepsis identification and monitoring of disease progression. Consequently, therapeutic strategies aimed at preserving or restoring a healthy microbial ecosystem may offer novel avenues for mitigating sepsis severity and improving patient outcomes.

### 3.2 Drivers of dysbiosis

Multiple factors synergistically drive gut dysbiosis during sepsis. First, broad-spectrum antibiotic use—while essential for infection control—non-selectively depletes commensal bacteria, impairing colonization resistance and creating ecological niches for multidrug-resistant or opportunistic pathogens (71). The application of various types of antimicrobial agents such as ciprofloxacin, imipenem, and vancomycin can lead to a decrease in the abundance of beneficial bacterial groups including *Streptococcus* and *Lactobacillus* (72, 73). Antibiotics can reduce the production of SCFAs and disrupt the differentiation of immune cells such as memory/effector T cells and Treg cells (74). Antimicrobial agents can also disrupt the colonization resistance of commensal microbiota, increasing the risk of infection by pathogens such as *Clostridium difficile* (75). A study colonized the gut of mice with ampicillin-resistant non-pathogenic *E. coli* and found that it not only enhanced its own resistance but also induced the migration of commensal bacteria to the intestinal mucosa and extraintestinal tissues (76, 77). Phages demonstrate unique anti-inflammatory and immunomodulatory potential in antimicrobial therapy, and their mechanisms of action have significant advantages compared to traditional antibiotics, but they still face challenges in safety validation such as difficulties in purity control and uncertainties in biological behavior. Therefore, in the treatment of sepsis, it is necessary to emphasize the regulation of microbial homeostasis, and the mature application of phage therapy still requires further exploration (78).

Second, septic shock often leads to systemic hypoperfusion (79), causing intestinal mucosal ischemia-reperfusion injury, epithelial hypoxia, mitochondrial dysfunction, and apoptosis, thereby disrupting the microbial niche. Catecholamine release exacerbates ischemia following vasoconstriction (80, 81), and localized ischemia promotes the proliferation of Gram-negative bacteria while altering bacterial iron metabolism (82, 83). Superoxide anions generated during reperfusion exacerbate intestinal epithelial oxidative damage (84), leading to disruption of tight junctions and cellular apoptosis, thereby increasing the risk of ectopic colonization by opportunistic pathogens.

Third, critically ill patients frequently experience gastrointestinal dysfunction or prolonged interruption of enteral nutrition, depriving fiber-fermenting symbionts of essential substrates and diminishing their metabolic activity (85). During septic shock, systemic vasodilation and capillary leakage (86) necessitate the use of vasopressor agents to elevate blood pressure, which subsequently reduces splanchnic blood flow and induces intestinal microcirculatory impairment (87, 88, 89). Ischemia and reperfusion injury compromise enterocyte energy metabolism and disrupt tight junction structure (90), facilitating the translocation of bacteria and endotoxins into the systemic circulation, while also dysregulating neurohormonal control of gastrointestinal motility, leading to delayed gastric emptying, diminished bowel sounds, and feeding intolerance (91).

In the management of septic shock, enteral nutrition strategies should adhere to an individualized approach, prioritizing hemodynamic stability (e.g., mean arterial pressure ≥ 65 mmHg) and restoration of splanchnic perfusion. Initiation with low-dose enteral nutrition is recommended over aggressive pursuit of nutritional targets, with gradual titration based on gastrointestinal tolerance. European guidelines recommend progressively increasing protein intake to 1.3 g/kg/day, whereas American guidelines support higher doses (1.2–2.0 g/kg/day), while cautioning against overfeeding which may exacerbate intestinal ischemia or microbiota dysbiosis (91).

Finally, stress-induced elevations in cortisol, catecholamines, and pro-inflammatory cytokines (e.g., TNF-α, IL-6) can directly suppress the growth and metabolic function of beneficial microbes, further reshaping the gut ecosystem from the host side (92).

Sepsis disrupts the intestinal barrier through multiple mechanisms, enabling PAMPs to more readily bind PRRs on host immune cells, triggering excessive release of pro-inflammatory cytokines such as TNF-α, IL-1β, and IFN-γ, concurrently activating the coagulation cascade and complement system, resulting in microthrombus formation and intestinal mucosal microcirculatory dysfunction (93, 94). This inflammatory storm initially manifests as systemic inflammatory response syndrome (SIRS), but subsequently induces an immunosuppressive state, creating a pro-/anti-inflammatory imbalance that compromises both innate and adaptive intestinal immunity, thereby promoting gut microbiota dysbiosis—characterized by a reduction in obligate anaerobes such as *Ruminococcus* and an increased proportion of conditional pathogens like *Proteobacteria* and *Enterococcus* (82). This establishes a vicious cycle of “barrier damage–dysbiosis–immune exhaustion,” ultimately exacerbating systemic inflammation, microcirculatory failure, and the progression of multiple organ dysfunction syndrome (MODS).

## 4 Pathogenic roles of gut dysbiosis in sepsis

Dysbiosis also mediates organ injury via gut–organ axes: the gut–lung axis exacerbates ARDS; the gut–liver axis promotes hepatocellular damage and bile acid dysregulation; and the gut–brain axis contributes to sepsis-associated encephalopathy via microbial metabolites crossing the blood–brain barrier. Metabolically, SCFA deficiency impairs mitochondrial function in enterocytes and disrupts immunometabolic reprogramming in immune cells. Additionally, translocated LPS activates endothelial cells, promoting coagulopathy and disseminated intravascular coagulation (DIC). Thus, dysbiosis is not a passive bystander but a central orchestrator of systemic inflammation, organ failure, and immune paralysis in sepsis.

### 4.1 Biphasic immune dysregulation

Gut dysbiosis actively contributes to sepsis pathogenesis through immune dysregulation, multi-organ crosstalk, and metabolic disruption. It drives a biphasic immune response: early hyperinflammation via PAMP/TLR4/NF-κB activation and late immunosuppression through Treg and myeloid-derived suppressor cell expansion. These PAMPs activate PRRs (e.g., TLR4) and downstream NF-κB signaling (93, 95), triggering a cytokine storm characterized by elevated levels of TNF-α, IL-1β, and IL-6 (96). This excessive inflammation induces endothelial dysfunction, vascular leakage, and tissue hypoperfusion, culminating in acute organ injury (94). As sepsis progresses, the host immune status transitions from an initial hyperinflammatory response to a state of immunosuppression. This process is characterized by comprehensive dysregulation of both the innate and adaptive immune systems. Neutrophil apoptosis increases (97), macrophages polarize toward an M2 anti-inflammatory phenotype (98, 99), and immature dendritic cells promote the differentiation of regulatory T cells and secretion of inhibitory factors such as IL-10 and TGF-β (100). During the early hyperinflammatory phase, increased intestinal permeability facilitates the translocation of bacterial products (e.g., lipopolysaccharide, LPS) into the systemic circulation. While regulatory Tregs become overactivated, disruption of Th1/Th2 balance, lymphocyte apoptosis, immune paralysis, and reactivation of latent viruses significantly increase the risk of secondary infections, contributing to immunoparalysis and heightened susceptibility to secondary infections (101, 102). Concurrently, myeloid-derived suppressor cells (MDSCs) expand dramatically (101, 103), further suppressing antimicrobial immunity. This immunosuppressed state is a key determinant of late-phase mortality in sepsis. Importantly, gut dysbiosis not only initiates this immune cascade but also sustains the imbalance throughout the disease course. A new paradigm suggests that pro-inflammation and immunosuppression may coexist rather than simply alternating temporally, with their intensity co-regulated by host genetic background, underlying diseases, and pathogen characteristics, ultimately leading to multiple organ failure through mechanisms such as microcirculatory dysfunction and mitochondrial impairment (104–106).

The PD-1/PD-L1 signaling pathway plays a central role in sepsis-induced immunosuppression, exacerbating immune dysregulation by inhibiting T cell activity, impairing myeloid cell function, and inducing immune cell apoptosis (107). Studies indicate that upregulation of PD-L1 expression delays neutrophil apoptosis, inhibits NET formation, and promotes Treg differentiation (108, 109). In early sepsis, myeloid-derived suppressor cells mediate T cell exhaustion via the PD-L1/PD-1 pathway, leading to a significant reduction in CD4+/CD8+ T cells (110). Clinical data confirm a positive correlation between PD-1 expression levels and disease severity/mortality^1111^, while anti-PD-L1 antibody therapy can restore immune cell function and improve prognosis.

Sepsis induces metabolic reprogramming via the mTOR/AMPK pathway, enhancing glycolysis; accumulated lactate inhibits monocyte/dendritic cell function through the GPR81 receptor, while disruption of the tricarboxylic acid cycle leads to abnormal accumulation of metabolites such as succinate and citrate, exacerbating inflammatory responses by mechanisms including HIF-1α stabilization and promoted prostaglandin synthesis (111). These metabolic disturbances are directly linked to energy metabolism defects in immune cells, revealing a metabolic-immune interaction mechanism that provides new therapeutic targets for sepsis.

In sepsis, multiple signaling pathways interact to drive immune dysregulation and organ damage (112). The NF-κB pathway, activated via TLRs, triggers transcription of pro-inflammatory cytokines (e.g., TNF-α, IL-1β), exacerbating systemic inflammation. The JAK/STAT pathway, activated by cytokines (e.g., IFN-γ, IL-6), regulates immune cell differentiation and inflammatory gene expression. The NLRP3 inflammasome, activated via a two-signal model (PAMPs/DAMPs priming and intracellular activation events), promotes caspase-1-dependent release of IL-1β and IL-18. HIF-1α is stabilized under hypoxic conditions and synergizes with NF-κB to amplify inflammation. The synergistic imbalance of these pathways constitutes the core pathology of sepsis, providing critical entry points for targeted therapy. Moreover, sustained inflammasome activation can trigger gasdermin-mediated pyroptosis, a lytic and highly pro-inflammatory form of cell death. This process not only amplifies the systemic inflammatory cascade through the release of intracellular danger signals but also directly compromises tissue integrity, contributing to endothelial dysfunction and organ injury. Thus, excessive pyroptosis represents a critical downstream effector mechanism linking inflammasome signaling to the tissue damage and immune dysregulation characteristic of severe sepsis (113–115).

### 4.2 “Leaky gut” and metabolite alterations

Dysbiosis directly compromises intestinal barrier integrity, leading to “leaky gut” syndrome. This enables pathogen-associated molecular patterns (PAMPs) to activate monocytes and macrophages via PRRs, triggering a cascade of inflammatory responses that exacerbate systemic inflammatory response syndrome (SIRS) and multi-organ dysfunction (116). Diamine oxidase (DAO) is a highly active intracellular enzyme mainly located in the mature enterocytes of the upper intestinal villi (117). Reduced DAO activity is commonly associated with intestinal mucosal injury, and circulating DAO levels correlate positively with intestinal permeability, making it a useful biomarker of gut barrier dysfunction (118). Concurrently, the metabolic capacity of the commensal microbiota is severely impaired. Key immunomodulatory and barrier-protective metabolites—especially SCFAs—are markedly reduced. SCFA deficiency diminishes energy supply to intestinal epithelial cells, impairs mucus production, and reduces IL-22 secretion. Notably, cytokines such as IL-13 and IL-22 enhance the transcription and expression of the tight junction protein claudin-2, thereby increasing paracellular permeability via the “pore pathway.” In contrast, pro-inflammatory cytokines like IL-1β and TNF-α upregulate myosin light-chain kinase (MLCK), activating the “leak pathway” (118, 119). These combined metabolic and structural disruptions create a vicious cycle that amplifies barrier failure and systemic inflammation, contributing significantly to sepsis progression and poor outcomes.

### 4.3 Gut-organ axes in multi-organ dysfunction

Dysbiosis propagates systemic inflammation via gut–organ axes, driving remote organ injury and MODS. In the gut–lung axis, circulating inflammatory mediators (e.g., IL-6, TNF-α) and microbial metabolites alter alveolar macrophage polarization and phagocytic function, disrupting pulmonary immune homeostasis and exacerbating acute respiratory distress syndrome (ARDS) (120). Wang et al. (121) first revealed that the gut microbiota metabolite succinate is a key mediator of the gut-lung axis. Intestinal ischemia-reperfusion upregulates succinate-producing bacteria, reduces succinate-degrading bacteria, and leads to the accumulation of succinate in lung tissue. Both clinical and experimental data have confirmed that succinate activates the PI3K/AKT/HIF-1α pathway through the SUCNR1 receptor, drives M1 polarization of alveolar macrophages, and induces apoptosis of alveolar epithelial cells, ultimately leading to acute lung injury. Xie et al. (122) innovatively proposed that memory γδ T17 cells derived from the small intestine after sepsis are the primary cellular source of the pulmonary IL-17 signaling pathway and serve as a key factor in post-sepsis pulmonary hyperinflammation and lung injury. After sepsis, activation of the pulmonary Wnt/β-catenin signaling pathway mediates the migration of memory γδ T17 cells from the small intestine to the lungs through the transcriptional regulation of the chemokine CCL1 by the transcription factor lymphoid enhancer-binding factor 1 (Lef1) in alveolar macrophages. Furthermore, the study found that dextrochloramine ketone (S-KT) has potential therapeutic prospects in the treatment of sepsis-induced acute lung injury; S-KT can inhibit the migration of small intestinal γδ T17 cells to the lungs by suppressing the pulmonary Wnt/β-catenin signaling pathway, thereby alleviating sepsis-induced acute lung injury.

In the gut-liver axis, gut-derived neutrophils migrate to the liver via the portal vein and activate hepatic macrophages (Kupffer cells) by releasing neutrophil extracellular traps (NETs), thereby driving sepsis-associated liver injury. To investigate the molecular mechanism by which NETs activate Kupffer cells, Murao et al. (123) discovered that neutrophils migrating to the liver via the portal vein activate the PAR-1 receptor on the surface of Kupffer cells through neutrophil elastase (NE) carried by NETs, promoting the polarization of Kupffer cells into a pro-inflammatory M1 phenotype and triggering the release of large amounts of inflammatory factors, ultimately leading to liver injury. When bile acid synthesis or regulation is abnormal, it disturbs the composition and function of the gut microbiota in reverse. In sepsis, dysregulation of the FXR/FGF-19 signaling pathway (124) leads to loss of control over its antibacterial properties. High concentrations of bile acids can directly inhibit the growth of commensal bacteria by disrupting bacterial cell membranes, inducing protein denaturation, and chelating essential metal ions (125), while simultaneously promoting the expansion of pathogenic bacteria. The gut microbiota mitigates this toxicity via bile salt hydrolase (BSH), which converts conjugated bile acids into their free forms (126). When this adaptive balance is disrupted, a vicious cycle of “bile acid dysregulation–microbiota dysbiosis” forms.

The gut–brain axis also plays a critical role in sepsis-associated encephalopathy. Microbial metabolites—including SCFAs, tryptamine, and indole derivatives—can cross the compromised blood–brain barrier or signal via the vagus nerve, activating microglia and triggering neuroinflammation, which manifests as cognitive impairment, altered consciousness, or even coma (127). Wu et al. (128) discovered that small intestine-derived γδ T17 cells migrate to the meninges after sepsis, release IL-17A inducing mitochondrial damage in microglia, activate the cGAS-STING pathway, thereby triggering complement C1q-mediated excessive pruning of hippocampal synapses, ultimately leading to cognitive dysfunction. This pathway elucidates a new mechanism by which gut immune cells remotely drive neuroinflammation, providing a precise therapeutic target for sepsis-associated encephalopathy (SAE).

### 4.4 Metabolic and mitochondrial dysfunction

Dysbiosis-induced metabolic disturbances profoundly impair cellular energy metabolism and homeostasis. At the intestinal level, depletion of SCFA-producing bacteria reduces butyrate availability, leading to mitochondrial dysfunction in enterocytes, decreased ATP production, and downregulation of tight junction proteins—thereby exacerbating barrier failure (96). Systemically, SCFA deficiency impairs immunometabolic reprogramming, hindering immune cells’ ability to adapt their metabolic phenotype to the dynamic inflammatory milieu, thus prolonging inflammation and delaying tissue repair (129). Additionally, dysregulation of microbiota-dependent pathways involving tryptophan and bile acids further disrupts host metabolic flexibility (130), establishing a microbiota-driven metabolic–immune axis that contributes to sepsis persistence.

Metabolic reprogramming represents a critical component of sepsis, exerting a profound impact on disease progression and patient outcomes (131). Sepsis-induced metabolic reprogramming refers to the process by which cells adapt to environmental stress by adjusting metabolic pathways (e.g., glycolysis, oxidative phosphorylation, fatty acid and amino acid metabolism) (132), characterized by immune cells preferentially switching to glycolysis for rapid energy production (analogous to the Warburg effect) even under adequate oxygen conditions (133), yet this leads to energy deficits and lactate accumulation, thereby exacerbating organ dysfunction (134). Key signaling pathways include the PI3K/Akt/mTOR pathway, which drives immune cell metabolic reprogramming by enhancing glucose uptake and glycolysis, supporting immune activation but potentially causing metabolic disturbances (135, 136), and the HIF-1α pathway, which orchestrates a shift from oxidative phosphorylation to glycolysis under hypoxic conditions (137, 138), promoting glycolysis but resulting in lactic acidosis and tissue damage (139).

Multiple studies have confirmed that the mechanisms of sepsis-related complications involve dysregulation of gut microbial components and metabolic pathways. Xie et al. (140) discovered that the tripeptide RKH produced by the probiotic *Akkermansia muciniphila* may alleviate organ injury by antagonizing TLR4. Chen et al. (141) found that phenylalanine metabolism is associated with acute kidney injury, while the pentose phosphate pathway is linked to sepsis-associated encephalopathy (SAE). Fang et al. (142) indicated that the microbiota-derived metabolite indole-3-propionic acid may prevent neuroinflammation in SAE. Xu et al. (143) revealed through multi-omics analysis that disturbances in lipid, amino acid, and glucose metabolism in the hippocampus are potential mechanisms in SAE. Jia et al. (144) confirmed that sepsis-induced myocardial dysfunction (SIMD) is associated with abnormalities in multiple metabolic pathways (e.g., AMPK signaling). She et al. (145) proposed a novel mechanism for SIMD involving lysine malonylation of the voltage-dependent anion channel 2, leading to mitochondrial dysfunction and ferroptosis. Chang et al. (146) found that differences in lipid composition in ARDS patients may originate from oxidative stress. These findings highlight the central role of metabolic reprogramming and microbial interactions in sepsis complications, providing new targets for organ-specific therapy.

In sepsis, mitochondrial dysfunction is a key driver of hyperlactatemia and organ failure. The study Nuyttens et al. (147) found severely impaired mitochondrial pyruvate metabolism in septic mice, primarily manifested as reduced activity of the pyruvate dehydrogenase complex (PDC), fundamentally due to thiamine (vitamin B1) deficiency leading to insufficient PDC cofactor thiamine pyrophosphate (TPP), thereby hindering pyruvate oxidation and promoting pyruvate conversion to lactate. Therapeutically, TPP supplementation significantly restored PDC activity, improved mitochondrial oxidative phosphorylation, reduced lactate levels, and ultimately increased survival.

Reactive nitrogen species (RNS) and reactive oxygen species (ROS) bursts inhibit mitochondrial respiratory chain complexes and generate highly toxic peroxynitrite (ONOO^–^), directly damaging mitochondrial integrity, and also suppress the PINK1/Parkin pathway, impairing mitophagy and leading to accumulation of damaged mitochondria. Furthermore, Bax/Bak-mediated mitochondrial outer membrane permeabilization (MOMP), mitochondrial permeability transition (MPT) pore opening, and GSDMs pore formation increase mitochondrial membrane permeability, triggering mtDNA leakage, which subsequently activates the cGAS-STING, TLR9, and NLRP3 inflammatory pathways, amplifying the systemic inflammatory response; coupled with a shift toward glycolysis exacerbating energy crisis, ultimately leading to multiple organ failure (148).

### 4.5 Coagulopathy and endothelial dysfunction

**FIGURE 1:**
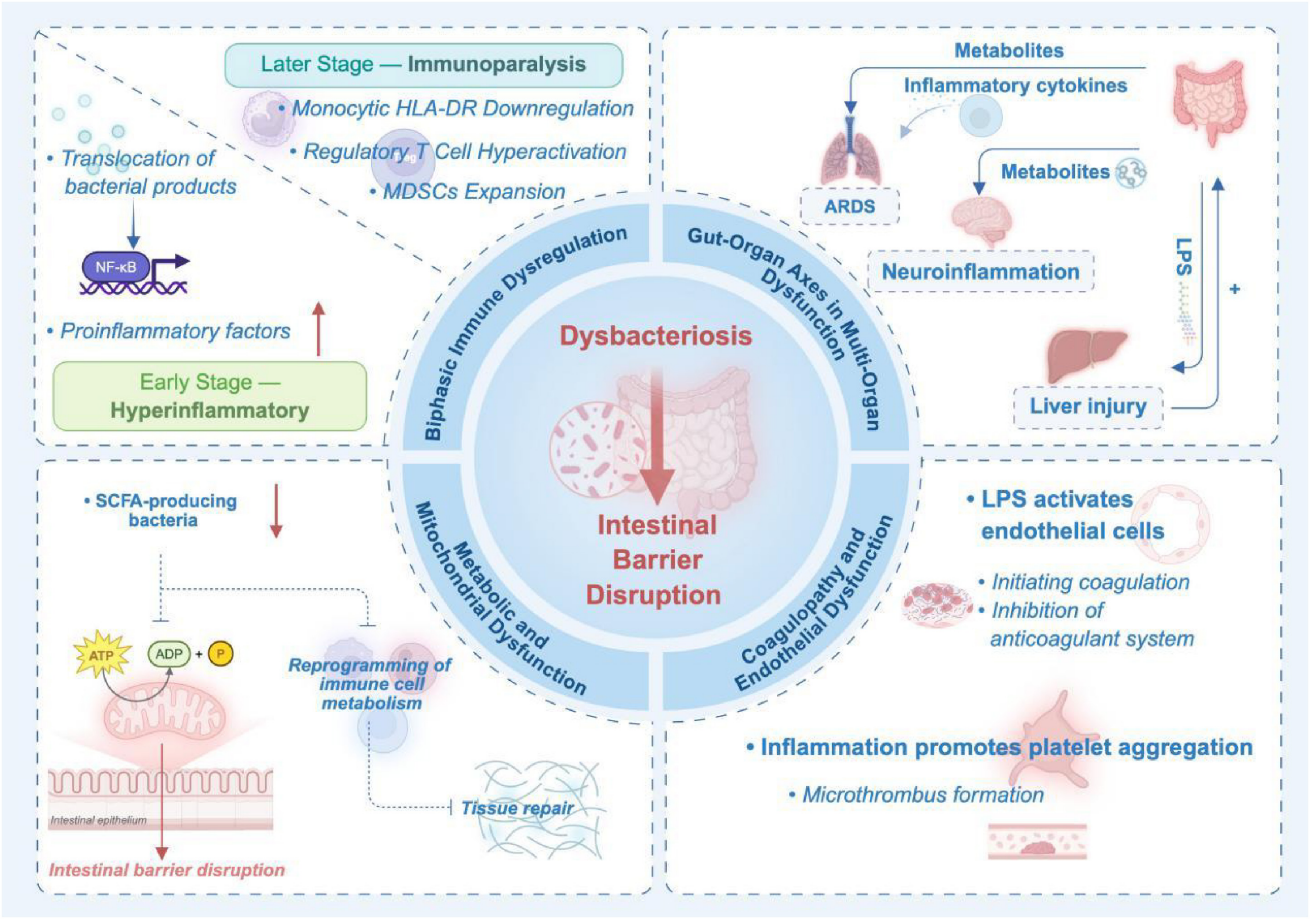
Pathogenic roles of gut dysbiosis in sepsis.

Sepsis-associated inflammation, immune dysregulation, and oxidative stress induce endothelial injury, microcirculatory failure, and a prothrombotic state (149, 150). Gut dysbiosis exacerbates this process: translocated LPS directly activates endothelial cells, inducing tissue factor expression and initiating coagulation, while simultaneously suppressing thrombomodulin and endothelial protein C receptor expression—key components of natural anticoagulant pathways (151). The resulting imbalance promotes platelet aggregation and microthrombus formation, culminating in disseminated intravascular coagulation (DIC) (152, 153) (Figure 1).

## 5 Microbiota as a source of prognostic biomarkers

Emerging evidence suggests that specific gut microbial signatures—such as enterotype classification or elevated Enterococcus abundance—may serve as predictive biomarkers for poor outcomes in ICU patients with sepsis (154). Future integration of metagenomics, metabolomics, and clinical data into multimodal predictive models holds promise for risk stratification and personalized therapeutic guidance.

The integrated application of multi-omics technologies has opened up new systemic research perspectives for sepsis biomarker discovery, shifting the field from single-marker exploration to multi-level integrative analysis. Genomics has revealed the impact of genetic susceptibility on sepsis outcomes, such as TLR4 gene polymorphisms (155). Transcriptomics has enabled molecular subtyping of sepsis through whole-blood gene expression profiling (156), while single-cell RNA sequencing technology has precisely identified novel cellular markers such as HLA-DRlowS100Ahigh immunosuppressive monocytes (157). Proteomics has constructed dynamic maps of the sepsis plasma proteome using high-throughput mass spectrometry, identifying protein clusters closely associated with organ injury, including prostaglandin D2 synthase and complement factors (158). Metabolomics has systematically elucidated metabolic reprogramming in sepsis, uncovering features such as amino acid metabolism dysregulation and abnormal lipid metabolism, for instance, the inverse correlation between cholesteryl ester levels and SOFA score (135, 159). The synergistic application of these technologies has not only achieved precise sepsis subtyping and mechanistic insights but also provided comprehensive molecular foundations for early diagnosis, prognosis assessment, and personalized therapy, though challenges remain in clinical translation, including high detection costs, complex data integration, and lack of standardization.

Artificial intelligence (AI) and machine learning (ML) technologies are driving sepsis biomarker research into a new era of precision diagnosis and treatment, significantly enhancing the efficiency of biomarker screening, model construction, and result interpretation by integrating multi-omics data, medical images, and clinical indicators. Prenosis Inc., (160) developed an AI-based Sepsis ImmunoScore tool integrating 22 parameters (e.g., PCT, respiratory rate), which achieved an AUC of 0.85 in the development cohort and received FDA approval as the first AI-assisted diagnostic system. Although China has also issued relevant expert consensus to promote technical standardization, most current studies remain focused on model training with insufficient external validation, and the models predominantly reveal correlations rather than causal relationships, limiting their clinical decision-support utility. Future efforts should strengthen the generalizability and clinical translation value of AI models through high-quality randomized trials and interpretability research (Table 1).

**TABLE 1:** Study on the association between gut microbiota and sepsis prognosis.

References	Key conclusions
Lu et al. (56)	α-HB-related targets could serve as promising therapeutic targets for sepsis management.
Ramalho Guerra et al. (58)	Gut bacteria can be potential biomarkers to characterize sepsis
Mikolas et al. (161)	Higher antimicrobial resistance burden in rectal samples of ICU sepsis patients is associated with longer survival time.
Yang et al. (162)	Propionate metabolism—derived from the gut microbiota’s short-chain fatty acid pathway—is significantly active in plasma cells of septic patients, with its metabolic level closely linked to immune regulation and disease progression, and a high-accuracy diagnostic model based on propionate metabolism–related genes has been successfully developed.
Gao et al. (163)	The gut microbiota, as a key modulator of immunity in sepsis, interacts with the host immune microenvironment in ways that—when integrated with multi-omics data via artificial intelligence—can generate a “combinatorial typing” biomarker framework capable of predicting patient outcomes, thereby enabling precision prognostication and personalized treatment in sepsis.
Xu et al. (164)	In patients with sepsis-associated acute kidney injury (SA-AKI), the gut microbiota is significantly linked to blood and urinary metabolites—particularly those involved in the lysine degradation pathway—suggesting that gut microbiota–driven metabolic dysregulation may contribute to SA-AKI development and thereby impact organ dysfunction and prognosis in septic patients.
Lee et al. (165)	Septic patients exhibit significantly reduced gut microbiome diversity, and a low Shannon index is independently associated with 6-month mortality, indicating that gut microbiota dysbiosis can serve as a key biomarker for predicting sepsis prognosis.

## 6 Microbiota-targeted therapeutic strategies and future perspectives

Several microbiota-targeted strategies show promise in sepsis management. Probiotics (e.g., *Lactobacillus*) and synbiotics can restore microbial balance and barrier function in preclinical models, though clinical results remain inconsistent. Fecal microbiota transplantation (FMT) restores diversity and SCFA levels in animal studies but faces safety and standardization challenges in critically ill patients. Nutritional interventions—particularly early enteral feeding with fiber, glutamine, and omega-3 PUFAs—support symbiont growth and mitigate inflammation. Antibiotic stewardship is essential to limit iatrogenic dysbiosis, while alternatives like phage therapy offer pathogen-specific control. Direct metabolite replacement (e.g., SCFAs) or modulation of microbial pathways (e.g., TLR4/NF-κB inhibition) presents a rapid therapeutic avenue. Future success hinges on integrating multi-omics data with AI to enable precision microbiome medicine tailored to individual sepsis phenotypes.

### 6.1 Probiotics and synbiotics

Probiotics, prebiotics, and synbiotics serve as important strategies for microbiota intervention in sepsis, aiming to alleviate antibiotic resistance and maintain intestinal microbiota balance by supplementing beneficial strains such as *Bifidobacterium* and *Lactobacillus* or prebiotics like inulin and fructooligosaccharides (166). In murine models, 4 weeks of *Lactobacillus* administration reversed sepsis-induced dysbiosis, reduced pathobionts, enriched beneficial taxa, and restored lipid and bile acid metabolism, thereby preserving barrier function, attenuating inflammation, and improving survival (167, 168). Engineered probiotics offer further innovation: a PEGylated *E. coli* strain engineered to overexpress IL-1 receptor antagonist (IL-1Ra) via outer membrane vesicles enhanced gut barrier integrity, suppressed systemic inflammation, and modulated pulmonary macrophage polarization, effectively mitigating gut–lung crosstalk-driven injury (169). Synbiotics, which combine probiotics and prebiotics, such as *Bifidobacterium breve* and *Lactobacillus casei* combined with galactooligosaccharides, can enhance immune responses and increase microbiota richness (170). Theoretically synbiotics can enhance probiotic engraftment and activity by providing selective substrates (e.g., dietary fiber) that promote SCFA-producing bacteria, offering a more robust approach to microbiota restoration (123, 171). Meta-analyses suggest that probiotics/synbiotics may reduce the incidence of sepsis, shorten ICU stays, and decrease mortality, but their effects are influenced by strain, dosage, and population heterogeneity (172). Despite promising preclinical results, clinical efficacy remains controversial, with inconsistent effects on overall mortality—likely due to variability in strains, dosing, timing, and patient heterogeneity.

In terms of efficacy, large-scale randomized controlled trials (e.g., the PROSPECT trial involving 2,650 mechanically ventilated patients) indicate that even common probiotic strains such as *Lactobacillus rhamnosus* GG or *Lactobacillus plantarum* 299v did not significantly reduce the incidence of ventilator-associated pneumonia (VAP), other infections, mortality, hospital stay, or antibiotic use (173, 174). This suggests that the “one-size-fits-all” intervention of traditional probiotics has limited clinical benefits in the complex environment of critical care (175).

Regarding safety, deep sequencing studies have confirmed that *Lactobacillus rhamnosus* probiotic formulations can translocate into the bloodstream in immunocompromised critically ill patients, causing bacteremia (176). In the PROSPECT trial, 12 patients tested positive for the bacteria in sterile sites. Probiotics may acquire or carry antibiotic resistance genes (e.g., through mutations), increasing the risk of resistant strain transmission (177). Additionally, critically ill patients often have impaired intestinal barrier function and immunosuppression, making them more susceptible to probiotic-related bloodstream infections (178).

Although probiotics have shown potential in some studies (e.g., trials in rural infants) to reduce respiratory infection, large-scale clinical trials (e.g., PROSPECT) have not confirmed their ability to reduce mortality or hospital-acquired infections in critically ill patients. The risks of bacteremia and antibiotic resistance gene transmission highlight the limitations of their efficacy due to population heterogeneity and raise safety concerns (179, 180). Next-generation probiotics, by exploring a broader range of commensal strains (e.g., *Lachnospiraceae*), can produce specific antimicrobial substances (e.g., lantibiotics secreted by *Blautia producta* strains that inhibit colonization by resistant bacteria) (181) and enhance intestinal barrier function and immune regulation through metabolites such as butyrate (182).

### 6.2 Fecal microbiota transplantation (FMT)

Fecal microbiota transplantation (FMT), the transfer of a healthy donor’s microbial consortium into a patient’s gastrointestinal tract, aims to restore a stable and functional gut ecosystem (183). While FMT demonstrates success rates exceeding 90% for recurrent Clostridium difficile infection and shows promise in preclinical sepsis models by improving survival and repairing the intestinal barrier (184, 185), its application in the complex, immunocompromised setting of sepsis remains highly investigational with significant safety concerns (186, 187).

Regarding the optimal timing, dosage, and route of administration for FMT in sepsis—a direct question posed by the editors—the current evidence base is insufficient to establish definitive protocols, but emerging insights and expert consensus offer preliminary guidance:

Timing: The theoretical window for FMT in sepsis is narrow and complex. Intervention too early, during the peak hyperinflammatory “cytokine storm,” could theoretically exacerbate systemic inflammation by introducing potent microbial-associated molecular patterns (MAMPs). Conversely, intervening too late, when the patient has entered a state of profound immunosuppression, may limit the therapeutic efficacy due to a diminished host capacity to support engraftment of new microbes. Preclinical studies suggest that FMT may be most beneficial when administered after the initial hyperinflammatory phase has been controlled with source control and antibiotics, but before the establishment of irreversible immune paralysis and multiple organ dysfunction. Some experts advocate for FMT as a “salvage therapy” for septic patients with persistent gut dysfunction or recurrent multidrug-resistant organism infections. However, high-quality data from human trials are critically lacking to define this optimal window.

Dosage: There is no consensus on a “standard” dose for FMT in sepsis. Dosing is typically based on donor stool weight or microbial cell count, extrapolated from experience in treating *C. difficile* infection. In critically ill patients with a severely disrupted gut ecosystem, a higher initial dose or repeated administrations may be necessary to achieve successful engraftment and overcome the dominance of pathogenic bacteria. The concept of a “minimum effective dose” for restoring ecological functions (e.g., butyrate production, colonization resistance) is an active area of research. Future strategies may move toward defined microbial consortia (next-generation probiotics) rather than whole stool, allowing for precise, reproducible dosing.

Route of administration: The optimal route remains debated and is likely patient-dependent.

Lower gastrointestinal route (colonoscopy/enema): Direct delivery to the colon allows for high-concentration engraftment in the distal gut. However, it poses procedural risks in hemodynamically unstable patients, including perforation and sedation-related complications.

Upper gastrointestinal route (nasogastric/nasoduodenal tube): This is less invasive and can be performed at the bedside, making it potentially more feasible for ICU patients. However, gastric acid and duodenal enzymes may reduce the viability of transplanted bacteria. Some evidence suggests this route may be associated with a higher risk of aspiration pneumonia in critically ill populations, a concern that is paramount in septic patients.

Oral capsules: This is the least invasive and most standardized approach, offering logistical advantages. However, gastric absorption and delayed, unpredictable release in a potentially dysmotile gut of a septic patient raise concerns about efficacy. Current formulations may also require a high pill burden.

In summary, while FMT holds theoretical promise, its application in sepsis is fraught with challenges. The current evidence does not support recommending a specific timing, dosage, or route. The significant safety risks, particularly the potential for transmitting drug-resistant organisms as tragically reported by the FDA and the risk of bacteremia in immunocompromised hosts, necessitate extreme caution (187). For now, FMT in sepsis should be strictly confined to clinical trials with rigorous protocols, including comprehensive donor pathogen and resistome screening, and should be avoided in hemodynamically unstable patients. Future research must prioritize establishing safety and then systematically evaluate these key parameters (timing, dose, route) in well-defined patient subpopulations to move this therapy from a promising concept to a viable clinical tool.

### 6.3 Nutritional interventions

Nutritional support is not only metabolic but also a key modulator of gut ecology and barrier function. Early enteral nutrition preserves mucosal integrity and prevents symbiont loss. Specific nutrients exert targeted effects: dietary fiber promotes the growth of *Bifidobacterium* and *Faecalibacterium*, enhancing anti-inflammatory and barrier-protective functions (188) and reducing antibiotic resistance gene prevalence (189). Glutamine—the primary fuel for enterocytes—supports epithelial repair and upregulates tight junction proteins (190). Omega-3 polyunsaturated fatty acids (PUFAs) modulate excessive inflammation, reduce complication rates, and improve clinical outcomes (191, 192). Personalized, microbiota-informed nutritional prescriptions may become integral to sepsis management. A randomized controlled trial, by comparing the effects of early application of total enteral nutrition (TEN), total parenteral nutrition (TPN), and supplemental parenteral nutrition (SPN) in septic patients, found from the perspective of intestinal microecology that TEN significantly improved nutritional indicators (prealbumin, albumin) and immune indicators (CD3+ T cells, IgG, complement C3), promoted the proliferation of beneficial bacteria such as *Enterococcus*, and showed an improving trend in short-chain fatty acids (SCFAs) such as acetate and propionate, while the TPN and SPN groups had limited effects (193).

Based on the Chinese “Guidelines for Medical Nutrition Therapy in Adult Sepsis Patients (2025 Edition),” early enteral nutrition (EN) serves as a key supportive measure for sepsis. The guidelines recommend that for septic patients who are hemodynamically relatively stable and have no gastrointestinal contraindications, trophic enteral nutrition should be initiated within 72 h to reduce ICU length of stay and duration of mechanical ventilation (194). The infusion method prioritizes continuous infusion to reduce adverse reactions in high-aspiration-risk patients, switching to intermittent infusion after intestinal function recovers (195). Additionally, early low-energy intervention (target energy within 70%) and appropriate protein supplementation (0.6–1.2 g⋅kg^–1^⋅d^–1^) can balance metabolic demands and organ load, ultimately improving intestinal microecology through multidimensional nutritional interventions (196–199).

### 6.4 Antibiotic stewardship and emerging alternatives

Given that broad-spectrum antibiotics are a major iatrogenic driver of dysbiosis, antimicrobial stewardship is critical. Current guidelines advocate for narrow-spectrum, short-duration, and pathogen-directed therapy to preserve core commensals. In this context, alternative strategies are gaining traction. Bacteriophage therapy offers high specificity against multidrug-resistant pathogens without disrupting the commensal microbiota (200, 201). Other emerging tools—such as antimicrobial peptides and precision microbiome editors—hold potential to control infection while maintaining microbial homeostasis.

The core of sepsis antibiotic administration management lies in timely initiation and precise selection. The SSC guidelines recommend initiating antibiotic therapy within 1 h after recognition of sepsis or septic shock (202), and delays exceeding 3–5 h significantly increase mortality (203, 204). However, evidence indicates that in non-shock patients, the risks of antimicrobial stewardship must be balanced (205).

Based on the 2022 Dutch Working Party on Antibiotic Policy (SWAB) guideline (206), the core of sepsis antibiotic management is precision and de-escalation strategy. It recommends selecting narrow-spectrum β-lactams (e.g., third-generation cephalosporins) covering potential pathogens based on the infection source, local resistance patterns, and individual patient risk (such as a history of colonization with resistant bacteria), avoiding routine combination therapy, and de-escalating within 48 h after obtaining susceptibility results. Emerging optimized approaches include pharmacokinetic/pharmacodynamic (PK/PD)-guided dosing (e.g., prolonged infusion of carbapenems to enhance efficacy), short-course therapy (4 days for postoperative intra-abdominal infection, 7 days for VAP), and biomarker (e.g., PCT)-guided discontinuation to reduce resistance. For penicillin-allergic patients, detailed allergy history assessment is used to evaluate cross-reactivity risk, prioritizing cephalosporins over alternatives such as fluoroquinolones, while strengthening therapeutic drug monitoring (TDM) to optimize vancomycin/aminoglycoside dosing, thereby balancing anti-infective efficacy and antimicrobial stewardship (AMS) goals.

Pathogen-specific coverage strategies include empirical therapy for patients at high risk of MRSA (e.g., recent surgery or immunocompromised), with guidance for discontinuation possible using nasal swab PCR screening (207, 208). Infections caused by multidrug-resistant Gram-negative bacteria require combination therapy (209). *Pseudomonas aeruginosa* coverage is suitable for patients with a history of hospitalization or structural lung disease (210). With increasing prevalence of ESBL producers, carbapenems are the preferred choice but require vigilance for resistance. High-risk patients (e.g., neutropenic) should be considered for antifungal therapy (211). The overall goal is to achieve early, targeted therapy through dynamic assessment, reducing resistance and adverse effects.

### 6.5 Metabolite replacement and pathway modulation

Since many microbiota effects are mediated by metabolites, direct supplementation or pathway modulation presents a rapid, bypass strategy. For example, inhibition of miR-155 attenuates sepsis-induced inflammation and barrier dysfunction by suppressing NF-κB signaling (113). The microbial dipeptide proline-leucine exacerbates acute lung injury via the C/EBP-β/NOD2/NF-κB axis (116), whereas mesenchymal stem cell–derived keratinocyte growth factor (KGF) ameliorates lung injury through the Gab1/ERK/NF-κB pathway (212). Similarly, the herbal polysaccharide from Radix Pseudostellariae alleviates septic liver injury by modulating gut microbiota and inhibiting TLR4/NF-κB signaling (213). Future integration of metabolomics with host response profiling may enable precision metabolite-based therapies.

The prospect of targeted therapy for sepsis lies in modulating immune imbalance and organ damage by intervening in key signaling pathways (e.g., JAK/STAT, PI3K/Akt, HIF-1α, etc.) (214, 215), with its core strategy shifting from the traditional “single-target inhibition” to “multi-pathway coordinated regulation.” Current research priorities include: using bioinformatics to screen multi-target drugs (e.g., heparin) (216), developing nanocarrier delivery systems to enhance targeting (217), neutralizing pathogen-associated molecular patterns (e.g., LPS) via monoclonal antibodies, and restoring immune homeostasis in combination with metabolic reprogramming (218).

**FIGURE 2:**
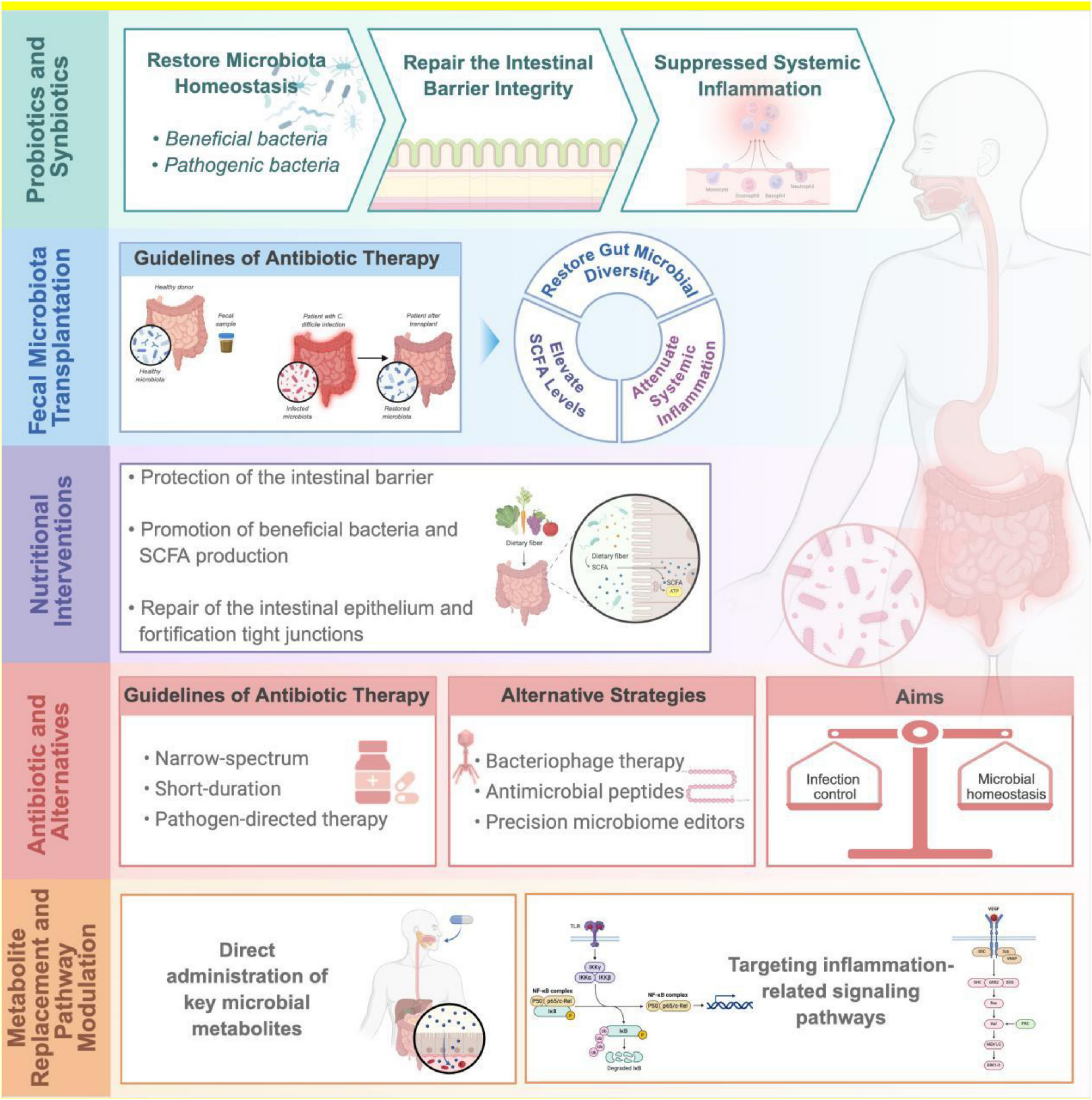
Microbiota-targeted therapeutic strategies and future perspectives.

However, clinical translation still faces three major challenges: heterogeneity in pathway activation due to individual differences, the difficulty in balancing drug selectivity and toxicity, and the optimization of patient stratification and intervention timing in clinical trials. Future breakthroughs will depend on individualized treatment strategies integrating multi-omics data, multi-target drug combinations (e.g., synergy between immune regulation and metabolic intervention), and novel technologies (e.g., organoid models, AI-assisted drug design) to accelerate the construction of a precision medicine system, ultimately achieving a paradigm shift from “disease control” to “organismal functional reconstruction” (Figure 2).

## 7 Prospective

The core role of gut microbiota in the pathophysiology of sepsis is increasingly well-defined, and related research is transitioning from associative description to mechanistic exploration and translational targeted intervention. Future advances and breakthroughs will depend on constructing a dynamic, integrated model of “host-microbe-environment” interactions and translating this knowledge into precise interventions that are both safe and scalable within the chaotic environment of intensive care.

Deeper integration and innovation are needed at the theoretical level. Sepsis research should move beyond viewing the gut microbiota merely as a passive victim or secondary factor, and instead position it as a key hub regulating infection response, immunometabolism, and inter-organ communication. Leveraging spatiotemporal dynamic analyses from multi-omics technologies—such as metagenomics, metabolomics, and single-cell transcriptomics—combined with animal models and organoid systems that better reflect clinical complexity, holds promise for meticulously deciphering how specific microbial communities and their metabolites precisely drive the chain reaction of biphasic immune dysregulation, metabolic reprogramming, and multiple organ dysfunction across different stages of sepsis and different clinical phenotypes. This requires focusing on how host genetic background, age, underlying diseases, and antibiotic administration interact with the microbiome to collectively shape the heterogeneous outcomes of sepsis.

Achieving the leap from “one-size-fits-all” to personalized therapy is the core objective of future clinical translation. This necessitates developing high-resolution biomarker systems based on the microbiome. By integrating metagenomic and metabolomic data, immune parameters, and clinical information, and utilizing artificial intelligence to build multimodal predictive models, early warning of sepsis risk, precise subtyping of the disease, dynamic monitoring of treatment response, and accurate prognosis prediction can be achieved. For instance, identifying specific “enterotypes,” resistance gene profiles, or metabolite profiles associated with immunosuppression or hyperinflammation will provide a direct basis for early intervention and personalized treatment selection.

Regarding treatment strategies, there is an urgent need to advance microbiome-targeted therapies toward greater precision and safety. The limitations of conventional probiotics in critically ill patients suggest that future live biotherapeutic products should shift toward screening “next-generation probiotics” with enhanced colonization resistance, barrier repair, or immunomodulatory functions, or utilize synthetic biology to design engineered bacteria for targeted delivery of therapeutic molecules. Fecal microbiota transplantation (FMT) requires the establishment of strictly standardized donor screening, preparation protocols, and safety monitoring systems. Nutritional support should evolve beyond universal guidelines into precise nutritional prescriptions based on individual microbiota profiles. Furthermore, directly supplementing or modulating key microbial metabolites (e.g., short-chain fatty acids, indole derivatives), developing precise phage therapies, and exploring microbiome editing tools are all promising new directions. Simultaneously, optimizing antimicrobial stewardship, implementing precise antibiotic de-escalation based on pharmacokinetic/pharmacodynamic principles and rapid diagnostics, remains the cornerstone for reducing iatrogenic dysbiosis.

Ultimately, translating these frontier concepts into reality hinges on close interdisciplinary collaboration and rigorous clinical validation. Microbiologists, immunologists, intensive care specialists, data scientists, and regulators must collaborate to design and conduct prospective, multicenter, large-scale randomized controlled trials that validate the efficacy and safety of microbiota-targeted interventions in well-defined patient subpopulations. Concurrently, exploring the cost-effectiveness of integrating these strategies into sepsis management pathways will provide comprehensive support for achieving precision medicine management of sepsis.

## 8 Conclusion

Sepsis, as a complex systemic disease, has a pathogenesis that extends far beyond a simple process of pathogen invasion and clearance. This review clarifies that the gut microbiota, functioning as a vast and dynamic “microbial organ” within the human body, has evolved from a digestive and metabolic partner into a critical “regulator” and “amplifier” in the pathophysiology of sepsis. Sepsis-induced gut microbiota dysbiosis, characterized by loss of diversity, depletion of beneficial commensals, and expansion of opportunistic pathogens, is not merely a passive consequence of the disease. Instead, it actively participates in and exacerbates a vicious cycle of systemic inflammatory response, organ dysfunction, and poor clinical outcomes by disrupting intestinal barrier integrity, driving biphasic immune dysregulation (early hyperinflammation and late immunosuppression), perturbing host energy and metabolic homeostasis, and propagating inflammation and injury via multiple gut-organ axes (e.g., gut-lung, gut-liver, gut-brain).

This recognition opens new dimensions for sepsis diagnosis and prognosis assessment, as gut microbial features and their metabolites show promise as valuable biomarkers. More importantly, it reveals novel intervention targets beyond conventional anti-infective therapies. Although existing microbiome-targeted strategies—such as probiotics, synbiotics, fecal microbiota transplantation (FMT), precision nutrition, and antibiotic stewardship—have demonstrated potential in preclinical studies and some clinical explorations to modulate the microbiota, mitigate injury, and improve outcomes, their clinical translation still faces major challenges including efficacy heterogeneity, safety risks, and lack of standardization.

Looking forward, overcoming the therapeutic dilemma in sepsis lies in a paradigm shift toward “precision microbiome medicine.” This requires a more systematic elucidation of the dynamic host-microbiota interaction networks in sepsis, refined patient stratification using multi-omics and artificial intelligence technologies, and the development and rigorous validation of personalized, dynamically adjusted microbiome-targeted integrated treatment strategies. By deeply integrating basic research, technological innovation, and rigorous clinical practice, the systematic incorporation of gut microbiota insights into critical care medicine will undoubtedly open new avenues for reducing sepsis mortality and improving long-term quality of life for patients.

## Data Availability

The original contributions presented in this study are included in the article/supplementary material, further inquiries can be directed to the corresponding author.
